# Global research on emerging trends of obstetrics during the COVID-19 pandemic: A bibliometric analysis

**DOI:** 10.1097/MD.0000000000039182

**Published:** 2024-08-02

**Authors:** Qin-Yu Cai, Yun-Ren Pan, Bei-Ning Deng, Wen-Dong Hu, Zong-Yan He, Xu Zhang, Wei-Zhen Tang, Tai-Hang Liu, Xia Lan

**Affiliations:** a Department of Obstetrics and Gynecology, Women and Children’s Hospital of Chongqing Medical University, Chongqing, China; b The Joint International Research Laboratory of Reproduction and Development, Ministry of Education, Chongqing Medical University, Chongqing, China; c Department of Bioinformatics, School of Basic Medical Sciences, Chongqing Medical University, Chongqing, China.

**Keywords:** bibliometric, CiteSpace, COVID-19, obstetrics, pregnant women, VOSviewer

## Abstract

Coronavirus disease-2019 (COVID-19) has caused continuous effects on the global public, especially for susceptible and vulnerable populations like pregnant women. COVID-19-related studies and publications have shown blowout development, making it challenging to identify development trends and hot areas by using traditional review methods for such massive data. Aimed to perform a bibliometric analysis to explore the status and hotspots of COVID-19 in obstetrics. An online search was conducted in the Web of Science Core Collection (WOSCC) database from January 01, 2020 to November 31, 2022, using the following search expression: (((TS= (“COVID 19” OR “coronavirus 2019” OR “coronavirus disease 2019” OR “SARS-CoV-2” OR “2019-nCoV” OR “2019 novel coronavirus” OR “SARS coronavirus 2” OR “Severe Acute Respiratory Syndrome Coronavirus-2” OR “SARS-COV2”)) AND TS= (“obstetric*” OR “pregnancy*” OR “pregnant” OR “parturition*” OR “puerperium”))). VOSviewer version 1.6.18, CiteSpace version 6.1.R6, R version 4.2.0, and Rstudio were used for the bibliometric and visualization analyses. 4144 articles were included in further analysis, including authors, titles, number of citations, countries, and author affiliations. The United States has contributed the most significant publications with the leading position. “Sahin, Dilek” has the largest output, and “Khalil, Asma” was the most influential author with the highest citations. Keywords of “Cov,” “Experience,” and “Neonate” with the highest frequency, and “Systematic Review” might be the new research hotspots and frontiers. The top 3 concerned genes included ACE2, CRP, and IL6. The new research hotspot is gradually shifting from the COVID-19 mechanism and its related clinical research to reviewing treatment options for pregnant women. This research uniquely delves into specific genes related to COVID-19’s effects on obstetrics, a focus that has not been previously explored in other reviews. Our research enables clinicians and researchers to summarize the overall point of view of the existing literature and obtain more accurate conclusions.

## 1. Introduction

The coronavirus disease 2019 (COVID-19) outbreak has had a profound and lasting impact on various aspects of society, including the economy, education, healthcare, transportation, and tourism.^[1–3]^ More importantly, up to November 31, 2022, there have been 638,175,811 confirmed cases of COVID-19, including 6612,970 deaths reported globally (source: https://covid19.who.int). Although WHO has claimed the end of the COVID-19 pandemic, and the mortality rate has reduced significantly, still new cases are reported with potential long-term complications appearance. As a predominantly respiratory illness, COVID-19 has a wide-ranging influence on various body systems. previous studies have shown that it could also affect other organs, such as the liver, kidney, and heart.^[4,5]^ Undeniably, as a world health threat not so long ago, the consequences of the health impacts of COVID-19 are numerous.^[6,7]^ It has been suggested that pregnant women and infants may be particularly susceptible to COVID-19 infection due to physiological changes in pregnancy involving the cardiopulmonary and immune systems, which may lead to an altered response to neo-coronavirus infections during pregnancy.^[8]^ COVID-19 poses risks to pregnant women, such as reduced thrombolytic capacity, resulting in thrombotic events,^[9]^ decreased respiratory function,^[10]^ various detrimental immune responses,^[11]^ and adverse pregnancy outcomes such as pre-eclampsia and preterm labor.^[12]^ Infants born to COVID-19-infected pregnant women are more likely to be infected with COVID-19 and have a higher incidence of adverse outcomes, including undernutrition, perinatal central nervous system damage, diabetic fetal disease, pulmonary atelectasis, delayed intrauterine growth, asphyxia, and other diseases and signs and symptoms.^[12–14]^ The number of articles examining COVID-19 has continued to rise since the outbreak of the coronavirus epidemic, and the impact of COVID-19 on maternal and neonatal outcomes has been substantial, resulting in a rapid growth of published literature in the field of COVID-19 and obstetrics. Therefore, we anticipate a growing focus and research interest in the intersection of COVID-19 and obstetrics. While there have been reports of intrauterine transmission of COVID-19, it appears to be rare, and there is still debate about vertical transmission and other outcomes.^[12,15]^

The Centers for Disease Control and Prevention, American College of Obstetricians and Gynecologists, and society for maternal-fetal medicine have issued guidance supporting the offer of COVID-19 vaccines to pregnant individuals, specifying that any currently authorized vaccines can be administered to pregnant women,^[16]^ which may be the most effective ways to protect against COVID-19.^[17]^ In the aftermath of the epidemic, there is increasing concern for the mental health of pregnant women, who are particularly vulnerable due to significant physiological and psychological changes.^[18]^ Previous studies manifested that pandemic-related concerns and the physical distance required for infection control may exacerbate mental health problems and loneliness. A majority of women had worries about the health of their relatives, children, and delivery plans.^[19]^ These psychological burdens are leading to a significant increase in depression, anxiety, and negative affect in non-pregnant women.^[20]^ Pregnant women are also experiencing other mental health conditions such as insomnia, post-traumatic stress disorder, and somatic symptoms.^[21,22]^

With the surge in publications across various disciplines, it is crucial to conduct a comprehensive review of the literature.^[23]^ Bibliometric analysis is an important method that can provide information on productivity, citations, and publication characteristics by providing statistical descriptions of publications.^[24]^ The application of bibliometric analysis in the medical field has garnered increasing attention, with a continuous rise in the publication of medical literature each year. Researchers seeking to ascertain research directions and current focal points within this extensive literature often consider employing bibliometric analysis. This method utilizes quantitative statistics to delineate the development of medical literature in a specific direction.^[25]^ It accomplishes this by examining various aspects: Firstly, concerning article publications, it quantifies the occurrence of keywords, citation counts, and citation relationships in the current year. Additionally, it tracks yearly article publications. Secondly, regarding authors, it identifies influential figures in a particular field and monitors their research trajectory by calculating indices like the H-index and M-index, among others.^[25,26]^ In essence, bibliometric analysis provides a holistic view of the knowledge structure within a research domain. This approach is particularly valuable for monitoring field trends, evaluating ongoing research outcomes, and forecasting future research areas. The term “knowledge structure” in this context refers to the construction of an understanding of various facets within a scientific field, such as prominent research directions, geographic concentrations of research, and influential institutions. It equips researchers with an initial grasp of a research area’s outcomes, influence, and configuration.^[27]^ Furthermore, bibliometrics serves as a robust tool for observing epidemiological studies, capable of swift execution at both national and international levels.^[28]^ To date, bibliometric analyses on COVID-19 have been conducted in areas concerning adult and pediatric populations, potential therapeutics (like essential oil therapy), and materials within the medical field. However, there hasn’t been a similar study specifically addressing pregnant women.^[29–31]^ Therefore, this study performed a bibliometric analysis of the literature on COVID-19 in obstetrics.

## 2. Methods

### 2.1. Research design

This study is a retrospective bibliometric analysis of COVID-19 and obstetrics. All metrics and experimental methodologies in this study adhere to the established guidelines of bibliometric analysis,^[32,33]^ including the application of clustering methods, ensuring the rigor and validity of the results to substantiate the discussion. The data utilized for this analysis is sourced from the Web of Science (WOS) and is collected following a meticulous search strategy. The specifics of this search strategy are detailed below.

### 2.2. Data sources

WOS is the world’s largest comprehensive academic information resource database, covering most disciplines, including more than 8700 core academic journals with the most influence in various research fields. A comprehensive online search was conducted on COVID-19-related studies in obstetrics in the Web of Science Core Collection (WOSCC) database from 1 January 2020 to 31 November 2022.

### 2.3. Search strategies

**Figure 1.**
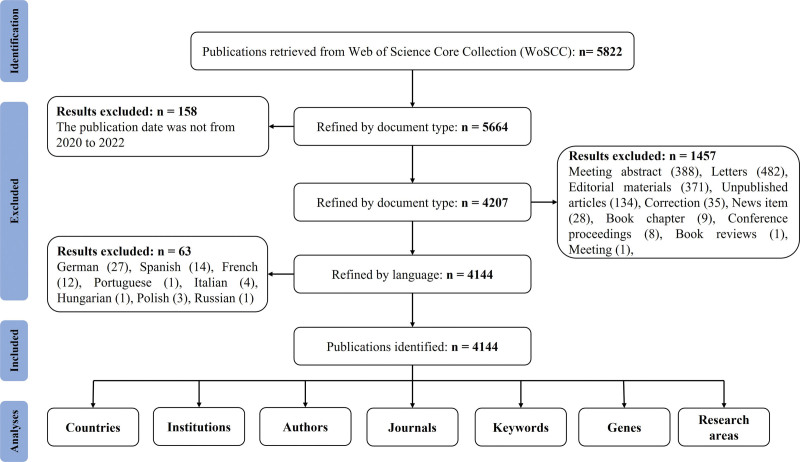
The flow chart of this study.

All literature searches were carried out in the Web of Science database. The search expression used to find the corresponding literature is: (((TS= (“COVID 19” OR “coronavirus 2019” OR “coronavirus disease 2019” OR “SARS-CoV-2” OR “2019-nCoV” OR “2019 novel coronavirus” OR “SARS coronavirus 2” OR “Severe Acute Respiratory Syndrome Coronavirus-2” OR “SARS-COV2”)) AND TS= (“obstetric*” OR “pregnancy*” OR “pregnant” OR “parturition*” OR “puerperium”))). The inclusion criteria are English articles; research papers published after peer review; reviews; articles published from January 1, 2020 to 30 November 2022; (5) articles retrieved from the Web of Science core collection. The exclusion criteria are non-English articles and publications other than original or review articles. Detailed information about the search strategies is shown in the flow chart (Fig. 1).

### 2.4. Data extraction

The data export function on Web of Science was used to export all the documents of search results in the plain text file of “Full Record and Cited References.” Then, the exported results were put into CiteSpace for de-reprocessing and getting the files for analysis.

### 2.5. Analysis

Web of Science was applied to analyze general characteristics of publications, including countries, institutions, articles, source publications, authors, and other information. Furthermore, impact factors and quartiles of categories were determined using Journal Citation Reports 2021. Microsoft Excel 2019 (Microsoft, Redmond), VOSviewer version 1.6.18 (Leiden University, Leiden, the Netherlands), and CiteSpace version 6.1.R6 64-bit (Drexel University, Philadelphia) was used for the presentation, analysis, and description of the data. Excel was used to present statistical analyses of data, including the visual representation of histograms in the study. VOSviewer was used to visualize the co-occurrence of keywords, authors, and countries.^[34]^ The project is represented by its label and a circle during network visualization. The label and size of the circle are determined by the weight of the item, and the larger the weight of the project, the larger the label and circle. CiteSpace was applied for clustering analysis, such as analyzing and visualizing co-citation networks, the type of cited articles, and articles citing others. It was also used for keyword clustering and to calculate centrality, revealing the comprehensive impact of the author in the field.^[35]^ To measure the citation impact and number of publications, we used R version 4.2.0 to calculate the total local citation score and total global citation score, the H-index, G-index, and M-index. The R version 4.2.0 was used to describe the visualization of co-country and co-institution, one three-field Plot (Sankey diagram) of the institution, author, keywords of COVID-19, and gravid. In the cooperation graph, the size of nodes is related to the number of publications and the thickness of the links is related to the strength of their cooperation relationships. The color of the node represents the year of the author’s publications. Furthermore, we analyzed gene and disease word frequencies using the “bibliometrix” package for bibliometric analysis in R version 4.2.0. The extraction of gene entity vocabulary from the literature was achieved by employing the pre-trained biomedical natural language processing model BioBRET, which is based on Python 3.11.10.

## 3. Results

### 3.1. Most productive and highest cited countries/areas

**Figure 2.**
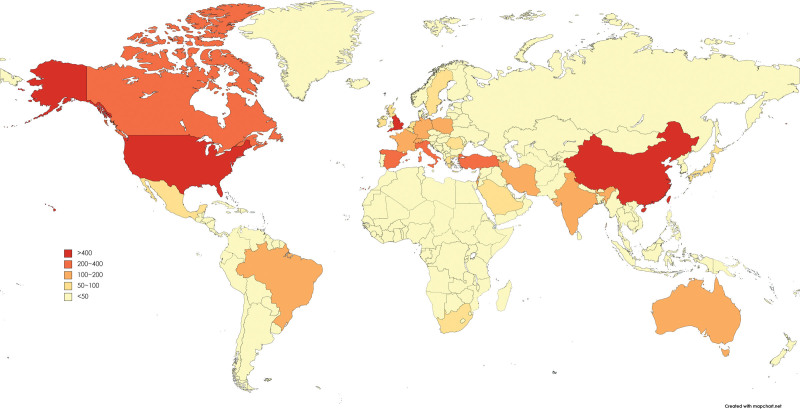
Distribution of countries or regions that have published articles. The heat world map is based on the number of articles. The publication volume of the article sets 6 gradients from high to bottom. The darker the color, the more articles are published.

The top 10 countries contributed 3713 articles. The USA contributed 1332 articles, accounting for 32.14% of the total, followed by England, which donated 423 articles, accounting for 10.21% of the total, afterward China, which contributed 416 articles, accounting for 10.04% of the total number of articles. Europe had 4 countries among the top 10, followed by 2 countries each from Asia and North America, and 1 each from South America and Oceania (Figure S1A, Supplemental Digital Content, http://links.lww.com/MD/N305). The top 10 countries with the highest number of published articles also rank in the top 10 in terms of citation (Figure S1B, Supplemental Digital Content, http://links.lww.com/MD/N305). The heat map displayed the distribution of countries or regions where papers were published, with most of the top 10 countries mentioned above appearing (Fig. 2).

### 3.2. Co-country of publications

Cooperative relations among the countries were analyzed, to which the top 50 articles were cited or appeared most frequently (Figure S2A, Supplemental Digital Content, http://links.lww.com/MD/N305). For instance, Italy tended to cite the United States, China, and Poland more often than it cited articles from these countries. On the other hand, it was cited more often by the United Kingdom, France, and Germany than it cited them. Following this, we utilized the gathered data to visualize the collaborative relationships between countries and institutions over the past 3 years. The top 3 countries in terms of publication numbers are the US, China, and Australia, with total link strengths of 529, 258, and 425, respectively. Likewise, the top 3 institutions in terms of publication numbers are the University of Queensland, the University of Colorado, and the University of Cambridge, with total link strengths of 193, 119, and 55, respectively (Figure S2B and C, Supplemental Digital Content, http://links.lww.com/MD/N305).

### 3.3. Analysis of institutions’ publications and funding agencies

The top 10 institutions with the most available articles contributed 1094 articles, accounting for 26.39% of the total number of articles published. The University of London published 181 articles, accounting for 4.37% of all articles, followed by Harvard University, which published 164 articles, accounting for 3.96% of all articles, and then the University of California system, which published 134 articles, accounting for 3.23% of all articles. The top 10 institutions with the most published articles are 4 in the United States, 2 in the UK, 2 in France, 1 in Canada, and 1 in China (Figure S3A, Supplemental Digital Content, http://links.lww.com/MD/N305); while the top 10 institutions with the most citations are 4 in the UK, 3 in China, 3 in the United States (Figure S3B, Supplemental Digital Content, http://links.lww.com/MD/N305). The funding agencies that published the most articles are also counted (Figure S3C, Supplemental Digital Content, http://links.lww.com/MD/N305).

### 3.4. Analysis of influential authors

The analysis of the ranked top 10 publication numbers of authors and cited authors showed that Khalil Asma from the St Georges Hospital in London is the author with the greatest centrality, citation count, and H-index (Table 1; Figure S4A, Supplemental Digital Content, http://links.lww.com/MD/N305). His publications were mostly systematic reviews, meta-analyses, and cohort studies. One of Asma’s articles, which reported on the clinical manifestations, risk factors, and maternal and perinatal outcomes in pregnant women with COVID-19, was cited 751 times. Sahin Dilek from Turkey has published the most research on COVID-19 and gravidas, but her publications did not have the same impact as Asma’s. A map of the authors was visualized to show their cooperative relationships, with a density of 0.0178 (Figure S4B, Supplemental Digital Content, http://links.lww.com/MD/N305). In addition, the author co-citation analysis identified the top authors with the greatest total link strength (Figure S4C, Supplemental Digital Content, http://links.lww.com/MD/N305).

**Table 1:** Top 10 authors and cited authors.

Rank	Author	Publication count	Centrality	H-index	TLCS	TGCS	Rank	Author	Citation count	H-index
1	Sahin, Dilek	34	0	13	21	3.25	1	Khalil, Asma	1459	16
2	Gyamfi-bannerman, Cynthia	27	0.08	14	27	3.5	2	Yang HX	987	4
3	Khalil, Asma	26	0.22	16	30	4	3	Li JF	965	5
4	Tanacan, Atakan	25	0	11	20	2.75	4	Zhang YZ	936	6
5	Edlow, Andrea G	22	0.02	10	23	2.5	5	Wang C	906	4
6	Goffman, Dena	16	0.01	10	21	2.5	6	Thangaratinam Shakila	867	9
7	Cetin, Irene	15	0.02	10	19	2.5	7	Galang RR	852	5
8	Prefumo, Federico	14	0.13	12	19	3	8	Guo JJ	837	3
9	Fell, Deshayne B	14	0	7	11	2.333	9	Chen HJ	836	3
10	Galang, Romeo R	13	0.01	5	13	1.25	10	Gilboa SM	834	6

Ranking: based on the number of total publications.

The University of Toronto has connections with high-publication authors, and results manifested that it has relationships with 7 authors in Figure S5A, Supplemental Digital Content, http://links.lww.com/MD/N305. Another three-field Plot is related to cited references, authors, and keywords (Figure S5B, Supplemental Digital Content, http://links.lww.com/MD/N305). This pot provides a visualization of which references had a large impact in this field and were cited by almost all the highly published authors.

### 3.5. Analysis of most active journals and research areas

To analyze journal distribution, we set a minimum publication threshold of 10 articles and visualized 60 journals (Figure S6, Supplemental Digital Content, http://links.lww.com/MD/N305). The *International Journal of Environmental Research and Public Health* and *BMJ Open* were found to have the strongest relationship, and the top 20 journals were manifested in this field by publications to concretize the data of the cross-citation network. The *International Journal of Environmental Research and Public Health* (IF = 4.614) had the most publications with 257 publications, while *BMJ Open* (IF = 3.007) ranked second with 173 records that were cited 776 times (Table 2).

**Table 2:** Top 10 most productive journals.

Ranking	Sources title	Output	Percentage (%)	JIF 2021	JCR quartile 2021
1	International Journal of Environmental Research and Public Health	134	3.234	4.614	Q2
2	BMC Pregnancy and Childbirth	114	2.751	3.105	Q1
3	International Journal of Gynecology Obstetrics	95	2.292	4.447	Q1
4	PLOS ONE	93	2.244	3.752	Q1
5	The Journal of Maternal-Fetal and Neonatal Medicine	91	2.196	2.323	Q2
6	American Journal of Perinatology	85	2.051	3.079	Q1
7	BMJ OPEN	74	1.786	3.007	Q1
8	Journal of Perinatal Medicine	74	1.786	2.716	Q2
9	American Journal of Obstetrics and Gynecology	69	1.665	10.693	Q1
10	Journal of Clinical Medicine	67	1.617	4.964	Q1

Ranking: based on the number of total publications.

The impact factor is an important index for evaluating journal influence. Among the top 20 journals, the *American Journal of Obstetrics and Gynecology* (IF = 10.693) ranked the highest, followed by *Frontiers in Immunology* (IF = 8.787) and the *Journal of Medical Internet Research* (IF = 7.077). Additionally, some articles analyzed in this study were from top-tier journals such as LANCET (IF = 202.731).

### 3.6. Characteristics of highly cited literature

Analysis of the top 10 most cited articles in the past 3 years was conducted on their citations and publications (Table S1, Supplemental Digital Content, http://links.lww.com/MD/N306). Most of the first 10 articles came from *LANCET, Circulation Research, the New England Journal of Medicine*, and other top journals with high quality. These articles focused on clinical topics such as hospitalization management, telemedicine, and maternal and child outcomes.

### 3.7. Analysis of keywords and related genes

Previous studies have suggested that analyzing keywords can predict future research trends and provide an overview of existing research.^[36,37]^ Using VOSviewer, we clustered keywords and found that “Cov,” “Experience,” “Systematic Review,” and “Neonate” were the most common keywords (Figure S7A, Supplemental Digital Content, http://links.lww.com/MD/N305). Interestingly, the frequency of most keywords steadily increased in 2021 and 2022, but the frequency of “Women,” “Pregnancy,” and “Impact” significantly increased in 2022 (Figure S7B, Supplemental Digital Content, http://links.lww.com/MD/N305), indicating that these 3 keywords are currently the most researched topics. Furthermore, investigations into associated genes have typically garnered the most extensive attention within the scientific community. Our analysis revealed that ACE2, CRP, and IL6, followed by TNF, TMPRSS2, CD4, and CD8A, have generated the highest level of interest among researchers in the relevant field (Figure S7C, Supplemental Digital Content, http://links.lww.com/MD/N305). These genes have been preliminarily confirmed to play a crucial role in the infection and diagnosis of COVID-19, making researchers more inclined to pay attention to how these genes affect the diagnosis of pregnant women.

### 3.8. Analysis of research areas

**Figure 3.**
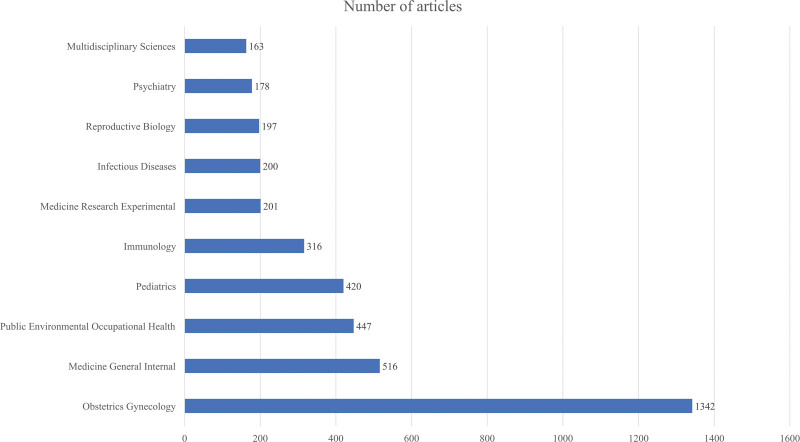
Using the Web of Science website, we made a bar chart based on the statistics of the top 10 disciplines involved in this research direction from January 2020 to November 2022.

The analysis of the research areas of the journal showed obstetrics gynecology, medicine general internal, and public environment occupational health were the top 3 research areas ranked by publication counts (Fig. 3). The research focused on preventive measures in obstetrics and gynecology, prenatal intervention, postpartum care, and drug research, among others. This result partly explains the current focus of research in this direction, given the challenges presented by COVID-19, particularly for vulnerable mothers.

## 4. Discussion

The COVID-19 pandemic has had a significant impact on our society, causing a global public health crisis. Since the initial report on obstetrics and COVID-19, there has been a notable increase in related literature.^[38,39]^ As our understanding of COVID-19 continues to advance, it is expected that the volume of such literature will persistently increase, providing more information on the obstetrician-related features, impacts, consequences, and therapeutic approaches of COVID-19.

A bibliometric analysis of countries/regions and institutions revealed that the top 10 countries with the most published articles were mainly located in Europe and America. The top 10 institutions with the number of articles published and cited were mostly concentrated in the United Kingdom and the United States, with China ranking third. Although China was the first country to discover and report COVID-19, the United States had the highest number of new coronavirus infections and deaths, resulting in a significant strain on their healthcare system.^[40]^ From March 2020, there was an exponential increase in SARS-CoV-2 infections in the United States.^[41]^ Lillie et al^[42]^ subsequently reported the first 2 cases of person-to-person transmission in the United Kingdom on February 28, 2020. European and North American countries invest more in research in this field, which contributes to their dominance in the published articles’ competence, output, and quality (https://www.nsf.gov/). The inter-country collaboration network analysis further demonstrates that the top 10 countries have a more active and intensive cooperation network, indicating the level of investment and collaboration among these countries.

Authors such as “Sahin, Dilek,” “Gyamfi-bannerman, Cynthia,” “And Khalil Asma,” were among the top authors in terms of the number of publications, illustrating their influential core group status in the field. However, the number of citations is not dominated by authors from Europe and North America. The most cited article, *“Clinical characteristics and intrauterine vertical transmission potential of COVID-19 infection in nine pregnant women: a retrospective review of medical records”* published in 2020, was authored by a Chinese researcher, and among the top 5 most cited authors, 4 are Chinese. This may be because the COVID-19 pandemic was first detected in China, allowing Chinese researchers to initiate investigation and research earlier, and their findings have been extensively cited in subsequent studies. The article “*Clinical characteristics and intrauterine vertical transmission potential of COVID-19 Infection in nine pregnant women: a retrospective review of medical records*,” published by Chen et al, was also ranked the first among co-cited articles in 2 bibliometric analyses of COVID-19 research.^[23,43]^ This clinical trial conducted in the early stages of the outbreak played a crucial role in subsequent research and became the cornerstone of research in this field. It is worth noting that no Chinese authors are among the top 10 authors in terms of the number of publications, indicating that COVID-19 research in China may focus more on infected patients than the association between obstetric diseases and COVID-19. However, pregnant women represent a uniquely vulnerable group in any infectious disease outbreak due to their altered physiology, susceptibility to infections, and compromised mechanical and immunological functions.^[7]^ Therefore, research on obstetrics and COVID-19 is essential. Evidence is accumulating that SARS-CoV-2 infection during pregnancy is associated with several adverse pregnancy outcomes, especially among pregnant persons with severe COVID-19 disease. In addition, the pandemic and its effects on healthcare systems have had adverse effects, such as increased stillbirths and maternal deaths on pregnancy outcomes.^[16]^ Hence, future research in this area is crucial.

Most of the articles on obstetric COVID-19 were published in general medical journals, including the *International Journal of Environmental Research and Public Health, BMC Pregnancy and Childbirth*, and *the International Journal of Gynecology Obstetrics.* This indicates that these journals may be good options for researchers looking to publish their manuscripts. The top 10 cited articles were mostly from prestigious journals such as *The Lancet, Circulation Research*, and the *New England Journal of Medicine,* which have a high impact factor and receive considerable attention in the field. Therefore, it is likely that future significant research findings in this area will be published in such journals. Researchers in related fields can focus on these 3 journals to learn about the latest findings and predict future research directions. These journals are also ideal platforms for future researchers to publish their results.

Analysis of keyword co-occurrence can identify the research directions and hotspots in a certain direction.^[44]^ As an emerging research field, the current prominent keywords continue to exhibit an upward trend, primarily centering around observational studies that emphasize outcomes, impacts, and risks on pregnant women. Mental health is also a significant focal point. The elucidation of the mechanisms of COVID-19 on pregnant women may unfold in subsequent investigations. The related keywords about the study mainly manifested “Cov,” “Experience,” and “Neonate,” while “Systematic Review” also garnered attention. This indicates that the research focus has shifted from clinical manifestations and diagnosis to studies summarizing the approach to the diagnosis and treatment of pregnant women with COVID-19. This is likely because our understanding of COVID-19 has deepened as the pandemic has continued, and updating and summarizing approaches to treating patients is crucial.^[45,46]^

Distinct from other bibliometric analyses, our research focuses on elucidating the mechanisms underlying the impact of COVID-19 on pregnant women. Notably, angiotensin-converting enzyme (ACE) 2 plays a pivotal role in the renin-angiotensin system and serves as the primary enzyme that interacts with COVID-19.^[47]^ Studies have reported the widespread expression of ACE2 in syncytiotrophoblast, cytotrophoblast, and endothelium in the placenta. This direct connection between COVID-19 and pregnancy may explain why ACE2 has become one of the most prominent genes of interest in this context. A recent study identified key genes, including ACE2, demonstrating that the drug ursodeoxycholic acid, used to treat liver disease, can block the ACE2 receptor and prevent the virus from entering cells.^[48]^ Moreover, inflammatory cytokines such as CRP, IL6, TNF, and CD4 play a significant role in investigating immune and pathological mechanisms.^[49,50]^ Research conducted in this field centers on examining the immune response and systemic inflammation in expectant mothers and newborns.^[51]^ Notably, some studies have explored the potential link between cytokines and COVID-19, including the relevance of CRP levels to the prognosis of pregnant individuals with COVID-19.^[52,53]^ The bibliometric analysis counts the most influential authors, journals, countries/regions, and institutions and shows some of their characteristics. During the early days of the COVID-19 outbreak, pregnant women with the virus experienced several pregnancy complications, such as fetal distress, premature rupture of membranes, preterm delivery, and stillbirth.^[54,55]^ As knowledge of the disease has deepened, various effective measures have been introduced to protect pregnant women, such as masks equipped with air filters and selecting the appropriate delivery method for infected women.^[56,57]^ Further studies have shown that the risk of death for infected pregnant women is similar to that of uninfected women, and there is no evidence that infected mothers are at high risk of miscarriage, preterm birth, abnormal amniotic fluid, cyanosis, or birth defects.^[58,59]^ Breastfeeding is a recommended option, ensuring there is no risk of respiratory transmission between mother and baby.^[60,61]^ In addition to these, people are also interested in the vaccination of pregnant women,^[16]^ equality of rights,^[62]^ and so on, and our understanding of these topics is gradually expanding. New research hotspots are continuously emerging, which will also influence the development of COVID-19 and obstetrics research in the future.

## 5. Limitations

False-positive and false-negative results may have affected the accuracy of our analysis. Furthermore, we only considered publications in the English language, which could have resulted in the exclusion of important non-English studies. Additionally, given the ongoing COVID-19 pandemic, the WOSCC database is continually updating, and our analysis may differ slightly from current findings.

## 6. Conclusion

In conclusion, our bibliometric analysis reveals that there has been significant research in the area of obstetrics and COVID-19 over the past 3 years, with the United States, England, and China being the primary countries, institutions, and authors with the most publications. The research focus has shifted towards the psychological and social effects of COVID-19 on pregnant women, with the top 3 subject areas being obstetrics and gynecology, public environment and occupational health, and general internal medicine. This study provides valuable insights into potential areas for collaboration and knowledge gaps in the field.

## Author contributions

**Data curation:** Qin-Yu Cai, Yun-Ren Pan, Wei-Zhen Tang.

**Formal analysis:** Qin-Yu Cai, Yun-Ren Pan.

**Funding acquisition:** Tai-Hang Liu, Xia Lan.

**Investigation:** Qin-Yu Cai, Wen-Dong Hu.

**Methodology:** Qin-Yu Cai, Bei-Ning Deng, Zong-Yan He, Xu Zhang, Wei-Zhen Tang.

**Project administration:** Qin-Yu Cai, Yun-Ren Pan, Zong-Yan He.

**Resources:** Qin-Yu Cai, Bei-Ning Deng, Wen-Dong Hu, Zong-Yan He.

**Software:** Yun-Ren Pan, Bei-Ning Deng, Wen-Dong Hu.

**Supervision:** Xu Zhang.

**Validation:** Wen-Dong Hu, Wei-Zhen Tang.

**Visualization:** Yun-Ren Pan, Bei-Ning Deng, Wen-Dong Hu, Xu Zhang.

**Writing – original draft:** Yun-Ren Pan, Bei-Ning Deng, Xu Zhang, Wei-Zhen Tang.

**Writing – review & editing:** Qin-Yu Cai, Zong-Yan He.

## Supplementary Material




