# Supplementary material for: Global research on emerging trends of obstetrics during the COVID-19 pandemic: A bibliometric analysis
Source: Medicine (Baltimore). 2024 Aug 2;103(31):e39182. doi: 10.1097/MD.0000000000039182 (PMC11296468; doi:10.1097/MD.0000000000039182)
Supplement: Supplementary file 1 [file medi-103-e39182-s001.docx]

**
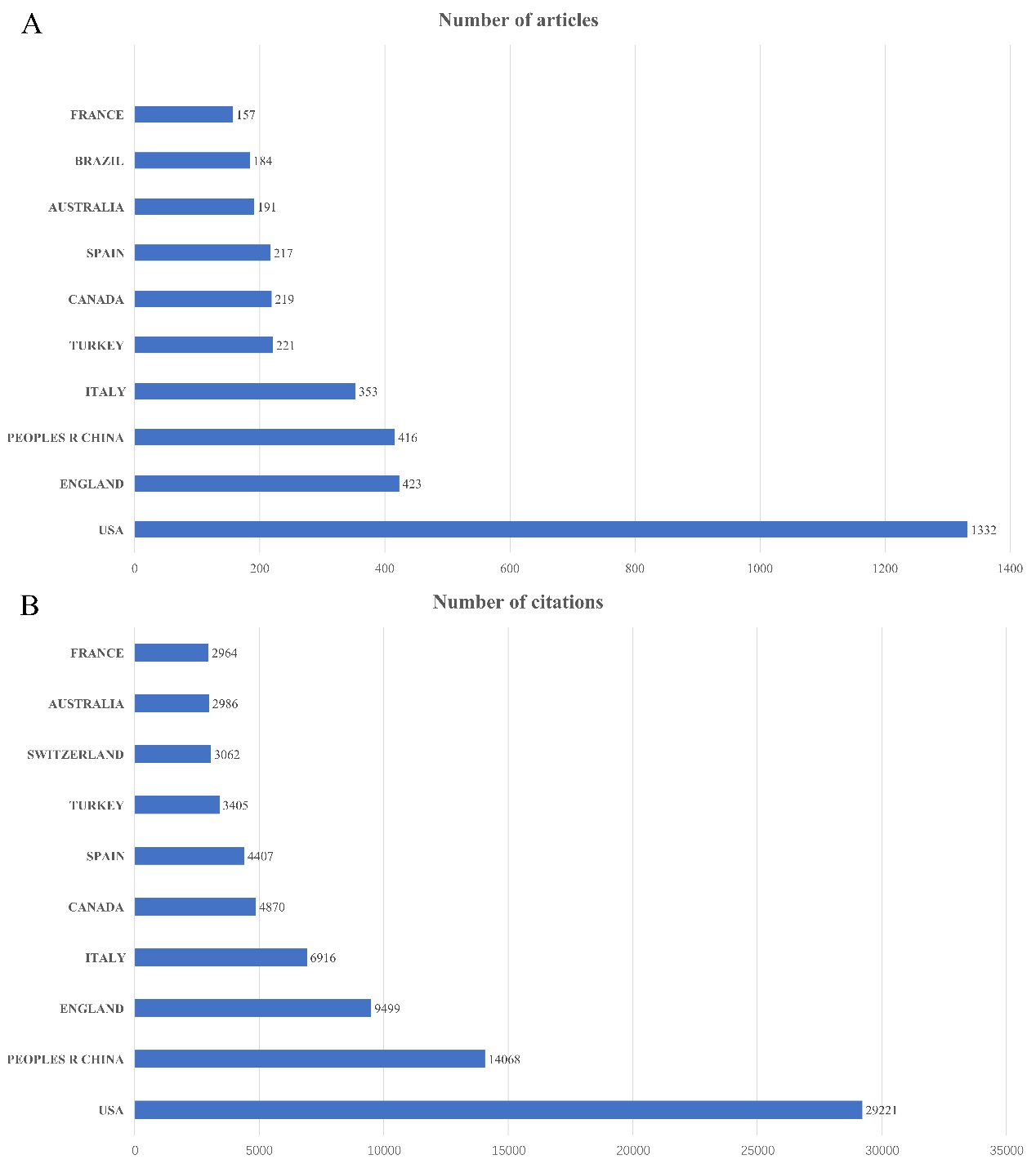
Figure S1. Top 10 countries with the most articles and the most citations.**

This bar chart is obtained by statistics of the top 10 countries with articles and citations from January 2020 to the end of November 2022. **(A)** The number of articles published in the top 10 countries. **(B)** The citations of each of the top 10 countries.

**
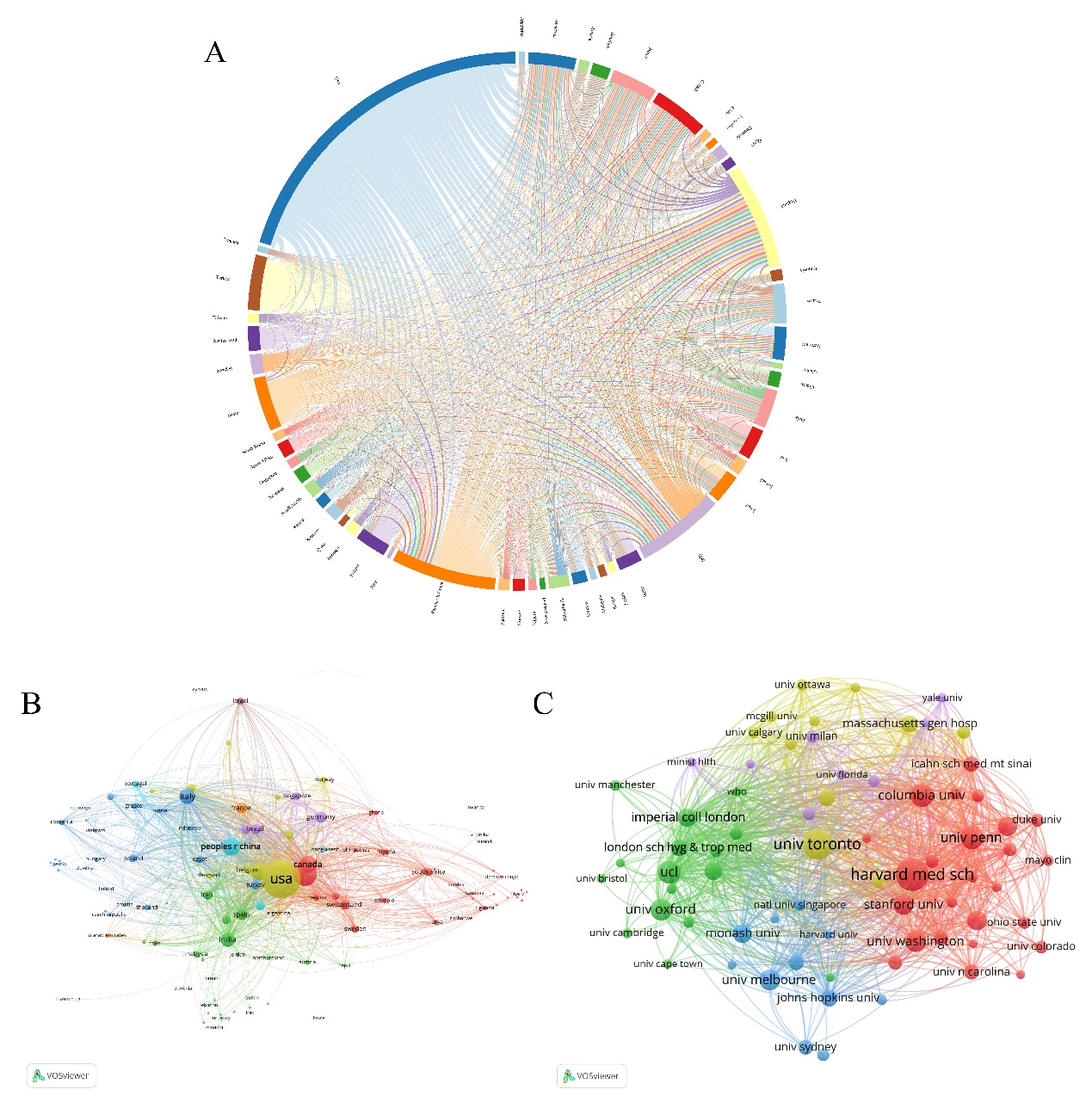
Figure S2. Co-countries and co-institutions in the field of COVID-19 and obstetrics. (**A) Chord diagrams show the cooperation between countries in the top 50 most cited articles from January 2020 to November 2022. **(B)** The cooperative network maps show the cooperative networks between countries that have made major contributions over the past two years. **(C)** The cooperative network maps show the cooperative networks between institutions that have made major contributions over the past two years.


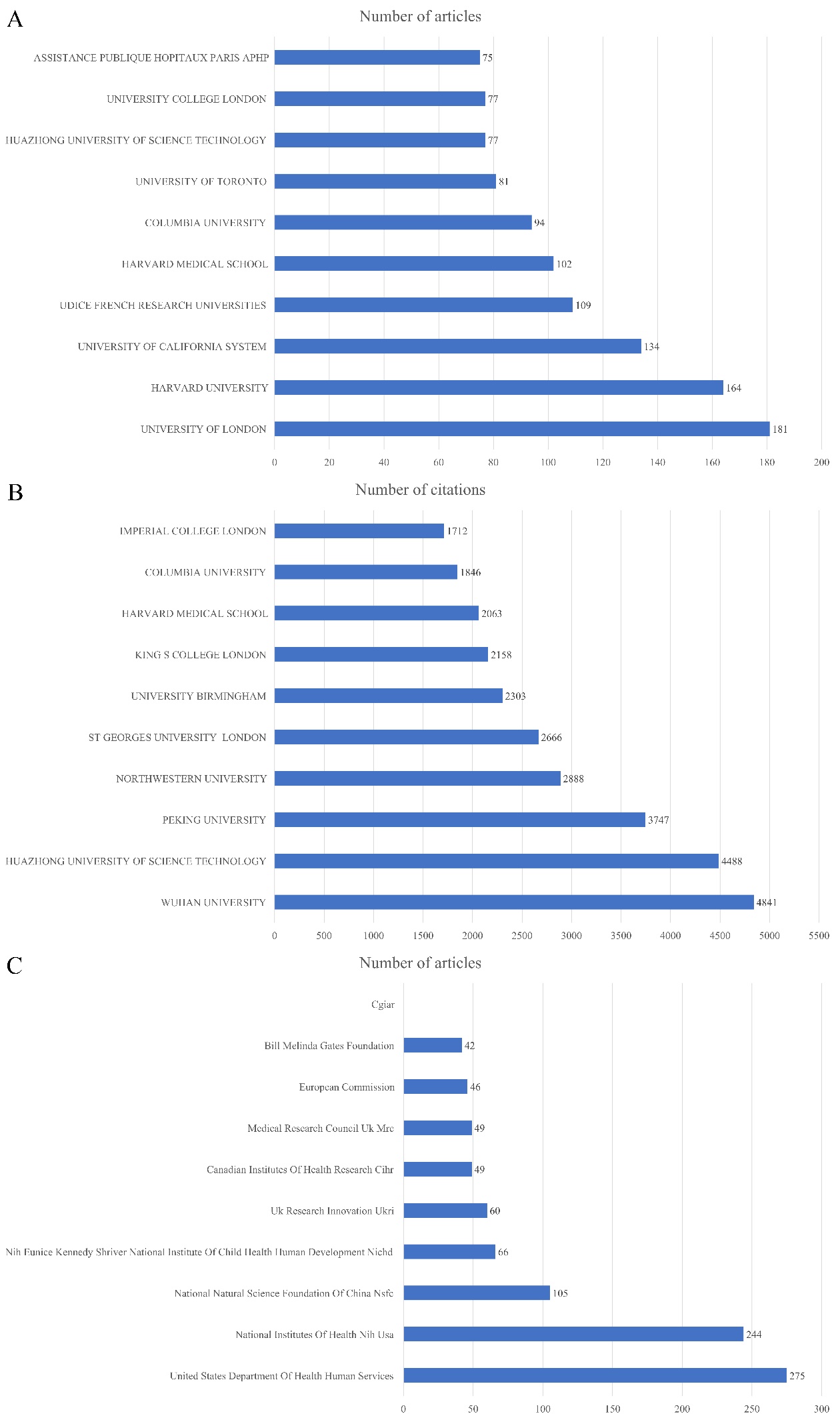
**Figure S3. Top 10 institutions and fund-funded organizations with the most articles and the most citations.** **(A)** The number of articles published in the top 10 institutions. **(B)** The citations of each of the top 10 institutions. **(C)** The number of articles published in the top 10 fund-funded organizations.


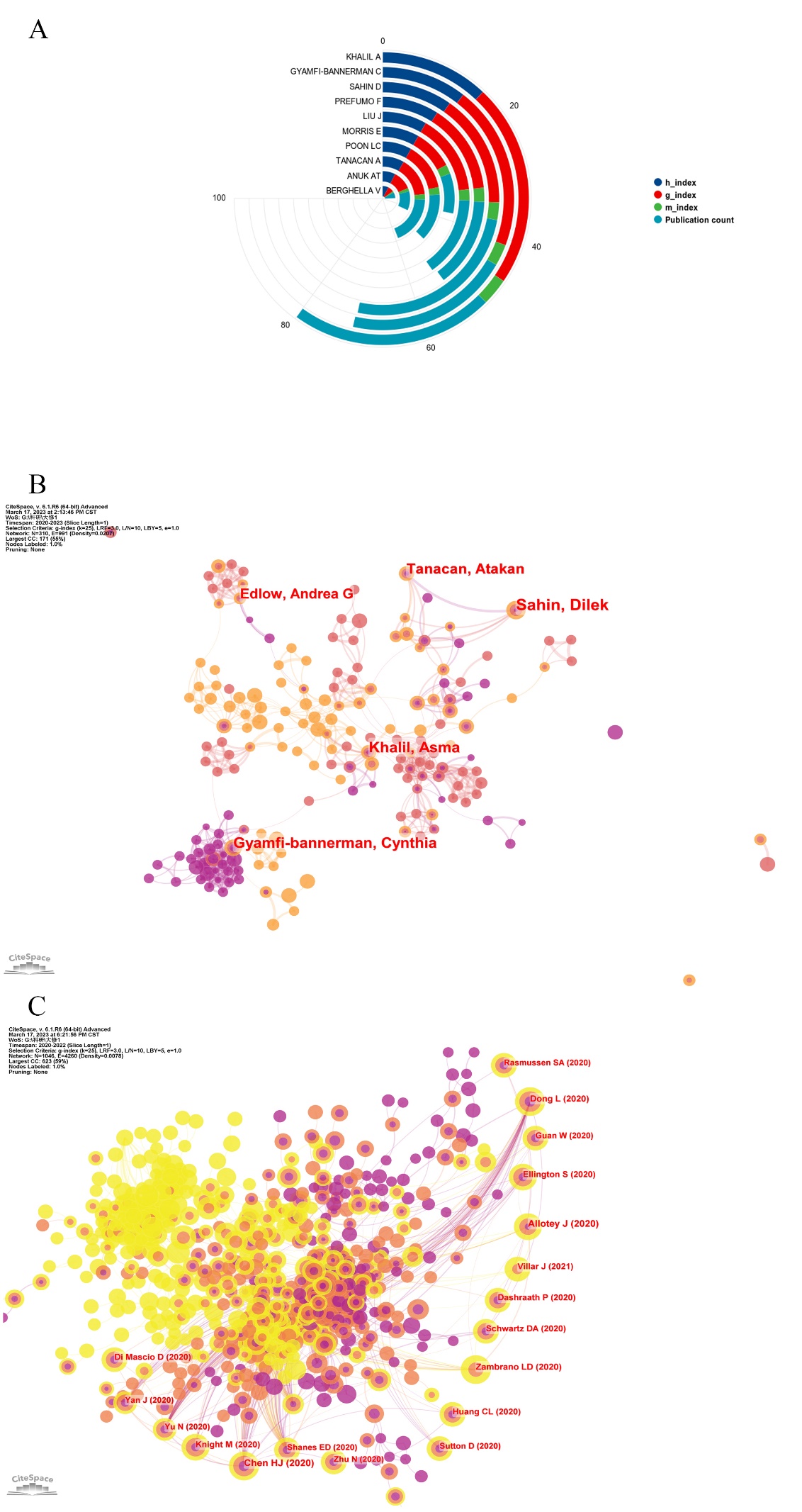


**Figure S4. Co-authorship analysis. (A)** A circular graph was obtained by adding the author's H index, G index, M index, and publication count. **(B)** Co-author map related to COVID-19 and obstetrics generated. **(C)** Co-reference map related to COVID-19 and obstetrics was generated.


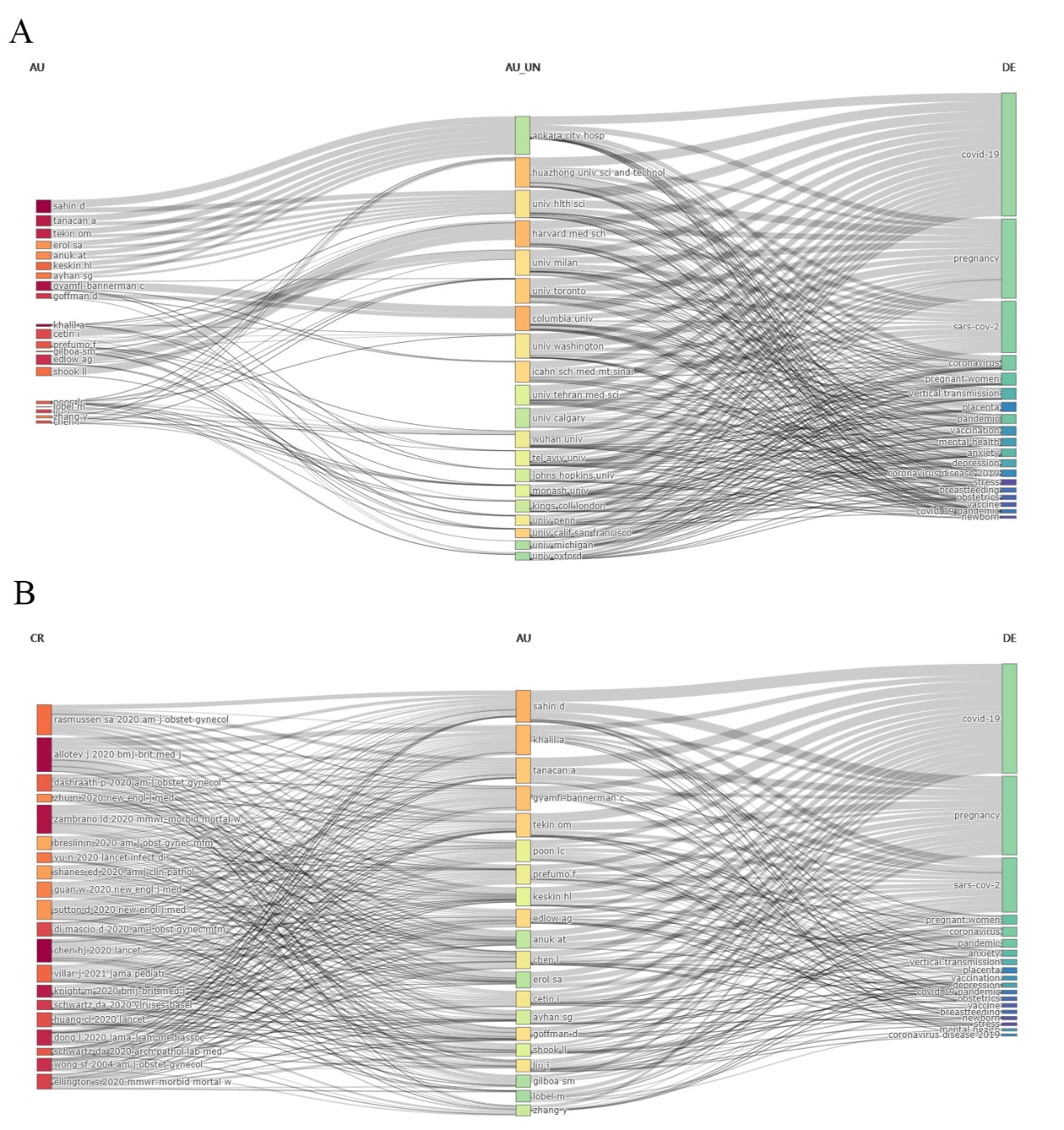
**Figure S5. Three-field Plot (Sankey diagram) of Cited references, Authors, and Keywords in the field of COVID-19 and obstetrics.**

**
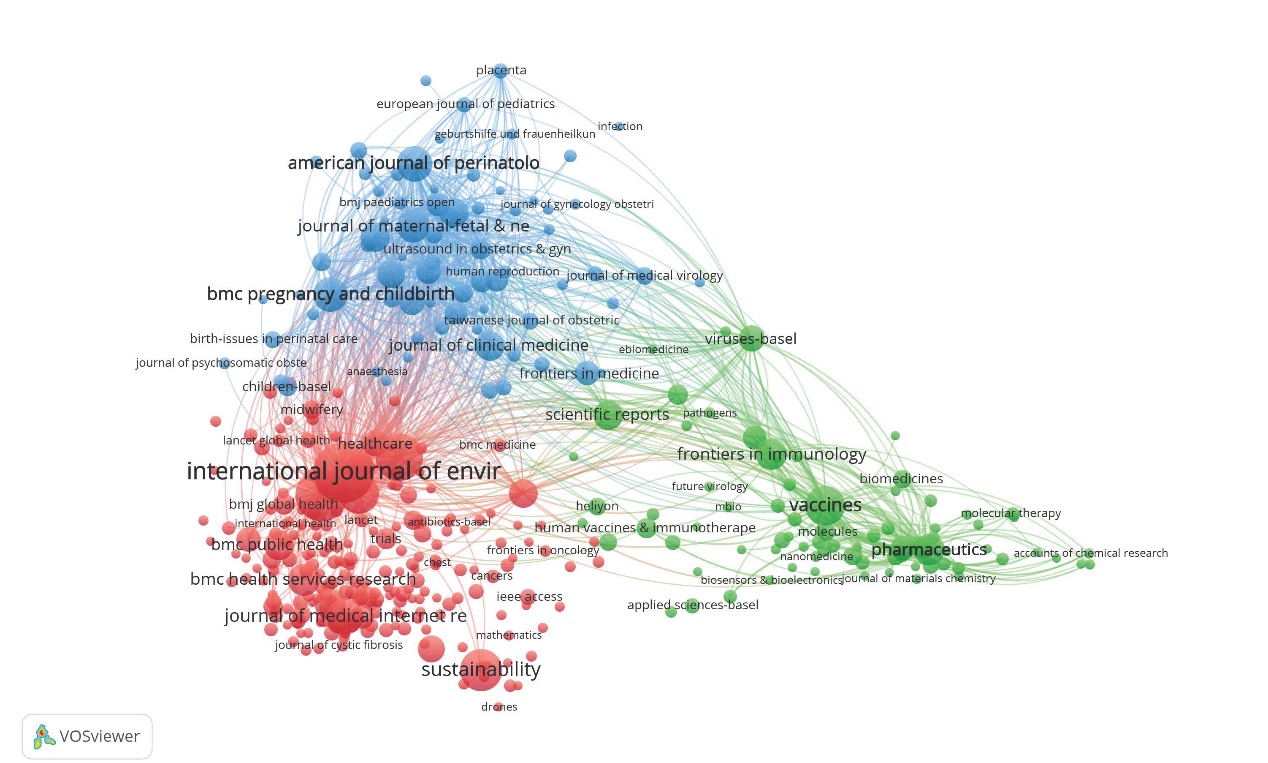
**

**Figure S6.** **Network visualization map of journal co-citation analysis generated by VOSviewer.**


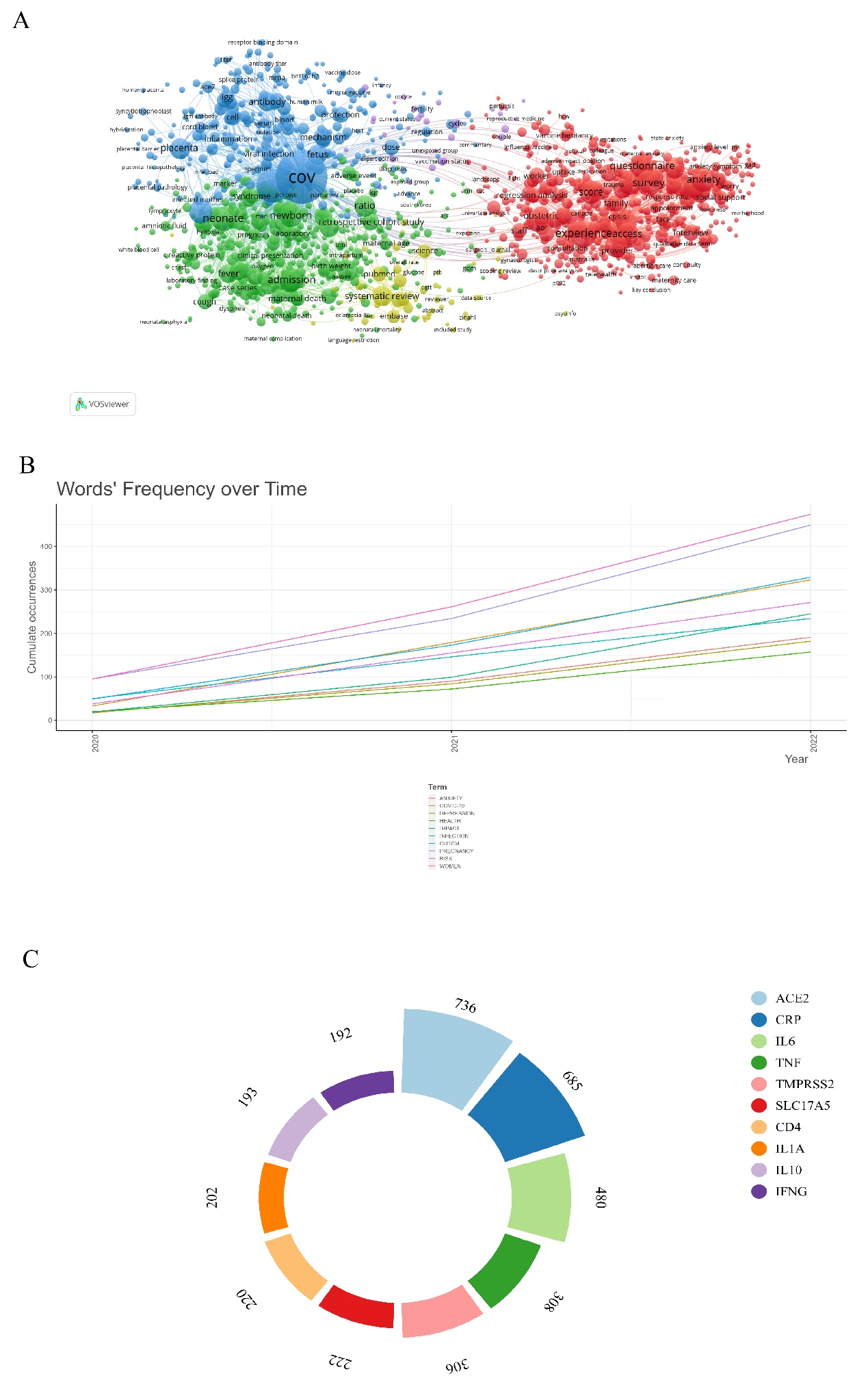


**Figure S7. The keywords and genes analysis.**

**(A) Cluster analysis of keyword co-occurrence map of COVID-19 and obstetrics. (B) The frequency change of keywords from 2020 to 2022. (C)The top 10 concerned genes in the area of obstetric COVID-19.**
