# Supplementary material for: Global research on emerging trends of obstetrics during the COVID-19 pandemic: A bibliometric analysis
Source: Medicine (Baltimore). 2024 Aug 2;103(31):e39182. doi: 10.1097/MD.0000000000039182 (PMC11296468; doi:10.1097/MD.0000000000039182)
Supplement: Supplementary file 2 [file medi-103-e39182-s002.docx]

**Table S1 Characteristics of the top 10 highly cited literature**

| **Ranking** | **Title** | **Total citations** | **Average citation frequency per year** | **Journal** | **References** |
| --- | --- | --- | --- | --- | --- |
| 1 | **Clinical characteristics and intrauterine vertical transmission potential of COVID-19 infection in nine pregnant women: a retrospective review of medical records** | **2,006** | **668.66** | **The Lancet** | **(**[**43**](#_ENREF_43)**)** |
| 2 | **Angiotensin-Converting Enzyme 2: SARS-CoV-2 Receptor and Regulator of the Renin-Angiotensin System Celebrating the 20th Anniversary of the Discovery of ACE2** | **982** | **327.33** | **Circulation Research** | **(**[**50**](#_ENREF_50)**)** |
| 3 | **Hospitalization and Mortality among Black Patients and White Patients with Covid-19** | **932** | **310.67** | **New England Journal of Medicine** | **(**[**51**](#_ENREF_51)**)** |
| 4 | **Clinical manifestations, risk factors, and maternal and perinatal outcomes of coronavirus disease 2019 in pregnancy: living systematic review and meta-analysis** | **769** | **256.34** | **British Medical Journal** | **(**[**52**](#_ENREF_52)**)** |
| 5 | **Telehealth for global emergencies: Implications for coronavirus disease 2019 (COVID-19)** | **754** | **251.34** | **Journal of Telemedicine and Telecare** | **(**[**53**](#_ENREF_53)**)** |
| 6 | **Telehealth transformation: COVID-19 and the rise of virtual care** | **636** | **212** | **Journal of the American Medical Informatics Association** | **(**[**54**](#_ENREF_54)**)** |
| 7 | **The Role of Chest Imaging in Patient Management during the COVID-19 Pandemic: A Multinational Consensus Statement from the Fleischner Society** | **612** | **204** | **Chest** | **(**[**55**](#_ENREF_55)**)** |
| 8 | **Assessing differential impacts of COVID-19 on black communities** | **606** | **202** | **Annals of Epidemiology** | **(**[**56**](#_ENREF_56)**)** |
| 9 | **How mental health care should change as a consequence of the COVID-19 pandemic** | **605** | **201.67** | **The Lancet**  **Psychiatry** | **(**[**57**](#_ENREF_57)**)** |
| 10 | **COVID-19 transforms health care through telemedicine: Evidence from the field** | **565** | **188.34** | **Journal of the American Medical Informatics Association** | **(**[**58**](#_ENREF_58)**)** |

Ranking: based on the number of total publications.
